# A commentary on: Does Living Donor Hyperoxia Have an Impact on Kidney Graft Function After Transplantation

**DOI:** 10.5812/numonthly.13621

**Published:** 2014-01-20

**Authors:** Mohammad Saeid Rezaee-Zavareh

**Affiliations:** 1 Students’ Research Committee, Baqiyatallah University of Medical Sciences, Tehran, IR Iran

**Keywords:** Data Interpretation, Statistical, Statistics, Nonparametric, Research Design

***Dear Editor,***

I read with great interest the valuable article entitled “Does living donor hyperoxia have an impact on kidney graft function after transplantation?” written by Dr. Rostami et al. (1). In this project, the authors have showed the effect of living donor oxygen therapy on function of grafted kidneys in recipients and for better comparison they’ve used a control group. Moreover, they also have assessed kidney function with some sound biomarkers such as neutrophil gelatinase-associated lipocalin, interleukin-18, and tumor necrosis factor-α that can be used for early diagnosis of kidney injury. Finally, they concluded that normobaric hyperoxia of living donors before kidney transplantation had no effect on kidney function in renal transplant recipients and suggested more studies in this field. There are only a few studies that investigated this effect in human (2-4) and I appreciate the admirable effort by authors for conducting this study; however, it seems that there are some remarks in their work that I want to point them out respectfully.

First, in the method part of this paper, it has just simply said that recipient patients were divided in two groups (case and control) but the difference between these two groups is not very clear. This important point can have impacts on some of the subsequent challenging points. Matching between case and control groups is a necessary part in such studies that can have important impact on the interpreting the result of a paper. Without an appropriate matching, we cannot determine one factor as responsible for the results of the study. In this paper, gender has a significant difference between case and control groups and there is not enough information about other factors that can be important in matching process like age, reason and duration of renal failure, and duration of hemodialysis. Another important point that should be taken into account is donors’ characteristics. There is not enough information about them especially those that had given kidneys to the control group. With regard to this point, it is necessary for readers of this paper to know about differences between these two groups of donors like age, gender, etc.

Additionally, according to the method part of this article, donors were exposed to oxygen for at least 2 hours for two consecutive days (before transplantation). After transplantation, the recipient patients were followed for two weeks. We know that in a cross-sectional study, we evaluate the association between the exposure factors (oxygen exposing in this study) and outcome (delayed graft function in this study) simultaneously. In addition to this follow up, there is an intervention (oxygen inhalation therapy) in their study. Again, we know there is not any intervention in an observational study like a cross-sectional. Therefore, three factors namely, randomization (it is not clear how exactly the recipients were randomized in two groups), intervention and follow up, make this study very powerful. Nevertheless, with consideration of these factors, I think the design of this project cannot be a cross-sectional type. In addition, we know these methodological mistakes can have some effects on selecting tests of the data analysis and therefor, they can influence on the results.

Ultimately, except gender of participants, the results of the paper contain no information about control group and comparison of it with case group. In addition, the only finding about case group that has been mentioned concerns the age but its importance is never mentioned in the discussion part. On the other hand, there are some analytical ways used in result part of this paper such as non-parametric correlation that are not reported in the method. In the other words, non-parametric test are not mentioned in the method part of the paper at all.
